# Management of dual traumatic arterial-venous fistula from a single shotgun injury: a case report and literature review

**DOI:** 10.1186/s12893-020-00833-5

**Published:** 2020-08-05

**Authors:** Rakan Nasser Eldine, Hassan Dehaini, Jamal J Hoballah, Fady Fayez Haddad

**Affiliations:** Division of Vascular & Endovascular Surgery, Department of Surgery, American University of Beirut Medical Center, PO Box 11-0236, Riad el Solh, Beirut, 1107 2020 Lebanon

**Keywords:** Traumatic, Arteriovenous fistula, Posterior tibial artery, Popliteal artery, Case report

## Abstract

**Background:**

Traumatic arteriovenous fistula (TAVF) is an uncommon vascular entity that arises in various locations, often from penetrating injuries, with a wide spectrum of signs and symptoms. This case report highlights the importance of suspecting multiple TAVFs after a single gunshot wound, especially if it involves pellets. It also sheds light on adapting treatment, whether endovascular or open repair, to the location and characteristics of each fistula.

**Case presentation:**

A 35-year-old male, with history of shotgun wound 5 months earlier, presented to our clinic with right lower extremity (RLE) edema and pain. Arterial duplex scan and subsequent angiogram showed two TAVFs at the popliteal and posterior tibial (PT) arteries, both of which could not be exactly localized with a computed tomography angiography (CTA) due to artifacts. The fistula connecting the posterior tibial artery (PTA) and vein was repaired endovascularly using a covered-stent, while the fistula between the popliteal artery and vein was repaired surgically. Postoperative follow-up at 3 months showed no arteriovenous fistula (AVF), patent vessels and distal stent stenosis at the PTA.

**Conclusions:**

Patients who sustain gunshot injuries with shrapnel or pellets and develop TAVF consequentially need to be followed up with the possibility of multiple AVFs in mind. Arterial duplex scan is highly sensitive to detect those AVFs, yet angiography remains gold standard, particularly with extensive metal artefacts. Endovascular repair, when feasible, should be considered first, unless the patient is unstable or has anatomical constraints that increase the risk of complications. Lastly, surgeons should be weary of deep venous thrombosis (DVT), the Branham effect and arterial aneurysmal dilation postoperatively.

## Background

An arteriovenous fistula (AVF) is an aberrant pathway between an artery and a vein. It can either be congenital or acquired [1]. Acquired AVFs are mostly iatrogenic, but they can be traumatic as well. Traumatic AVFs (TAVFs) constitute up to 3.9% of vascular injuries [2] and have been mentioned in a multitude of case reports and series. However, TAVF remains a challenging pathology due to the absence of official guidelines in the midst of variable presentations, multiple locations and different treatment modalities. In this paper, we report an interesting case of a 35-year-old male who acquired two TAVFs of the lower extremity, one of which was treated via an endovascular approach, while the other required an open surgery. This case report is written in accordance with the CARE guidelines [3].

## Case presentation

A 35-year-old male, previously healthy, presented to our clinic with swelling of his right thigh associated with pain of 4 weeks duration. The patient’s history was pertinent for a shotgun injury to his right lower extremity (RLE) 5 months prior to presentation, which had involved numerous pellet injuries to his popliteal artery, posterior tibial artery (PTA), and tibioperoneal (TP) trunk. His injuries had required multiple direct arterial and venous repairs, in addition to four compartments fasciotomies that were closed with skin grafts. Additionally, he had developed posttraumatic neuropathic pain and mild RLE weakness, which were improving and being followed up by neurology. During the interval time, patient was doing well until 4 weeks prior to this presentation.

Upon physical examination, patient had a marked swelling of his proximal RLE and a bruit on auscultation distally. He had an absent posterior tibial (PT) pulse, normal dorsalis pedis (DP) pulse and decreased range of motion (ROM) at the ankle. There were no signs of limb ischemia nor venous hypertension.

**Fig. 1:**
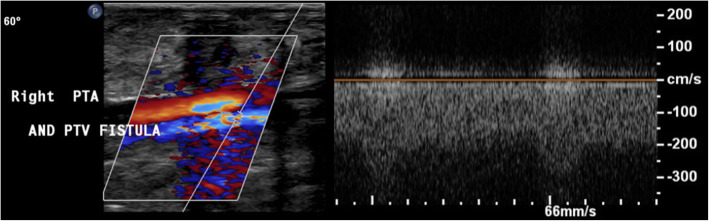
Arterial duplex scan showing an AVF between right PTA and PTV

**Fig. 2:**
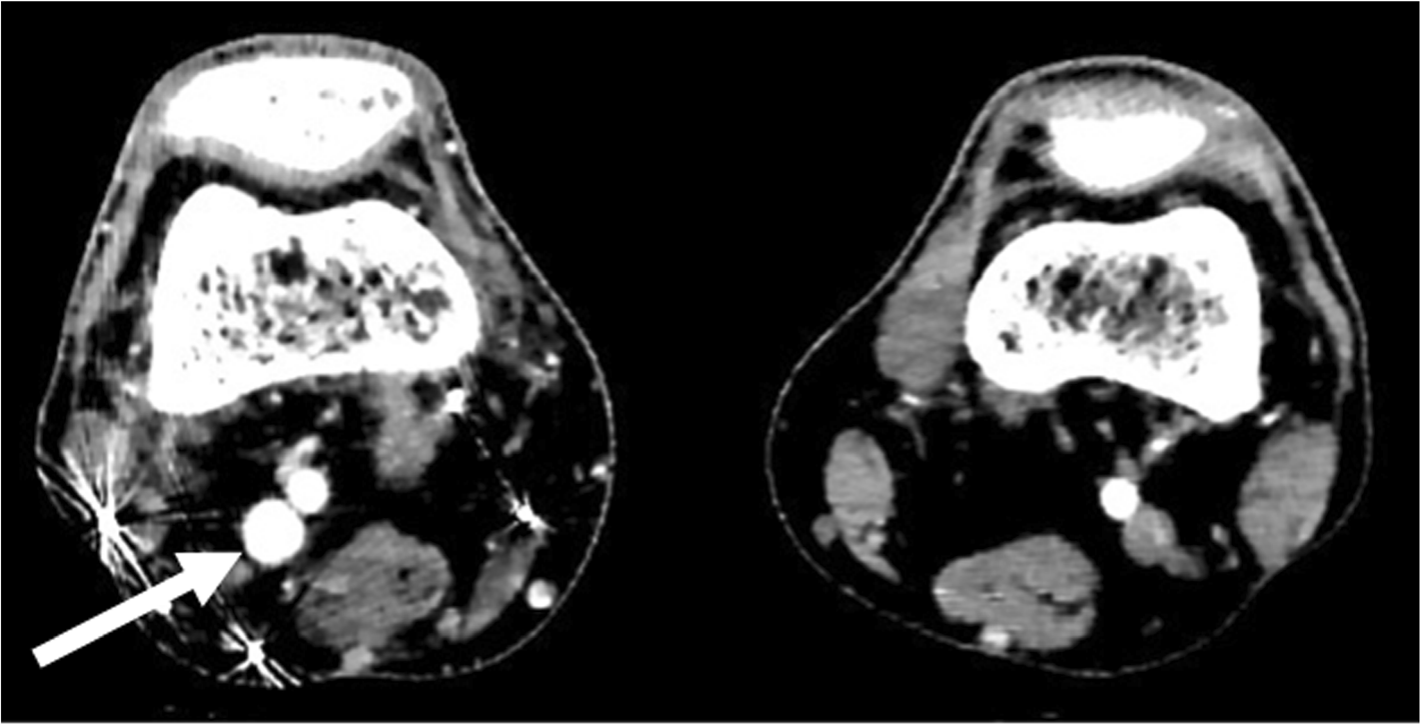
CTA showing early filling of right popliteal vein, indicating the presence of an AVF. Pellet artifacts in the posterior-lateral distal thigh

Arterial duplex scan showed high diastolic flow in the PTA and an arterialized flow in the posterior tibial vein (PTV). A reversed flow in the distal PTA was also noted. Indeed, a communication was eventually found at the level of mid-to-distal leg between PTA and PTV (Fig. 1). Computed tomographic angiography (CTA) showed early filling of the right popliteal and superficial femoral veins (SFVs), as well as dilated superficial veins of RLE, but could not identify the location of the AVF due to multiple pellets artifacts (Fig. 2). However, the brisk and substantial filling of the femoral vein on the arterial phase raised the possibility of a more proximal fistula than the distal leg. Angiography was planned with intension to treat depending on findings.

The right common femoral artery (CFA) was accessed and arteriography was done to confirm the previously diagnosed AVF between PTA and PTV. However, there was an additional distal popliteal AVF at the level of the tibial tuberosity. The decision was to offer surgical repair for the popliteal AVF; however, for the PTA one, an endovascular option would be done because of the extensive scarring from the initial exploration, in addition to the split-thickness skin graft at the fasciotomy sites.

**Fig. 3:**
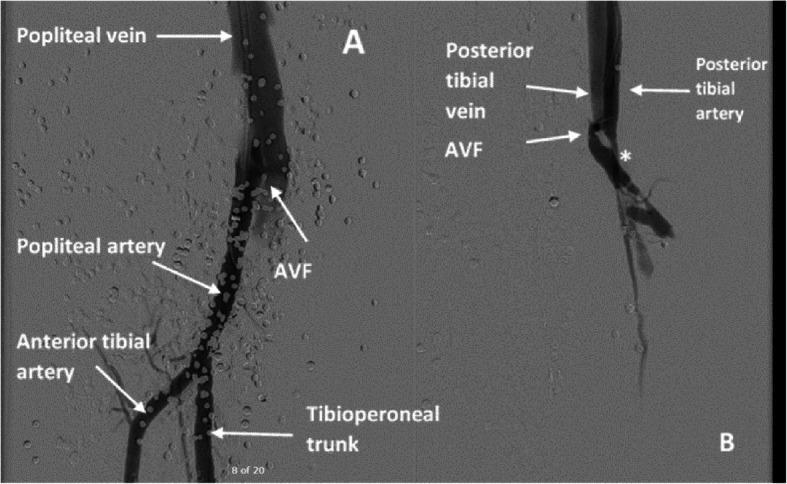
**a** Angiogram showing an AVF between popliteal artery and vein. **b** Angiogram showing an AVF between PT artery and vein. Post-AVF stenosis is demarcated with an *.

**Fig. 4:**
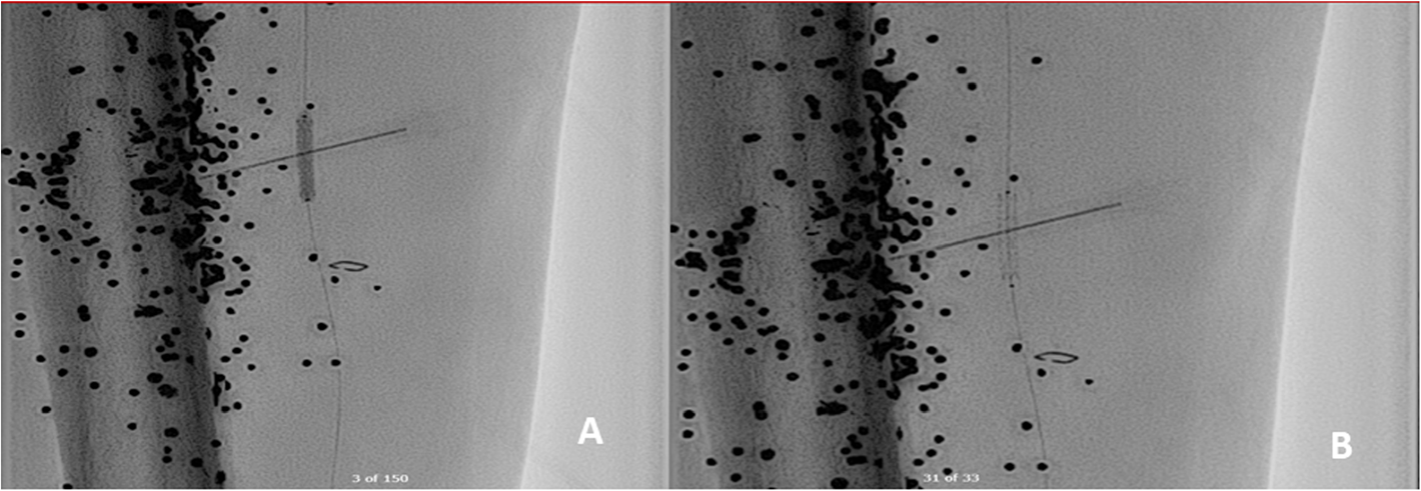
**a** A 3 × 15 mm coronary balloon used for pre-dilatation in PTA. **b** A 3 × 18 mm covered stent in the PTA at the level of AVF

**Fig. 5:**
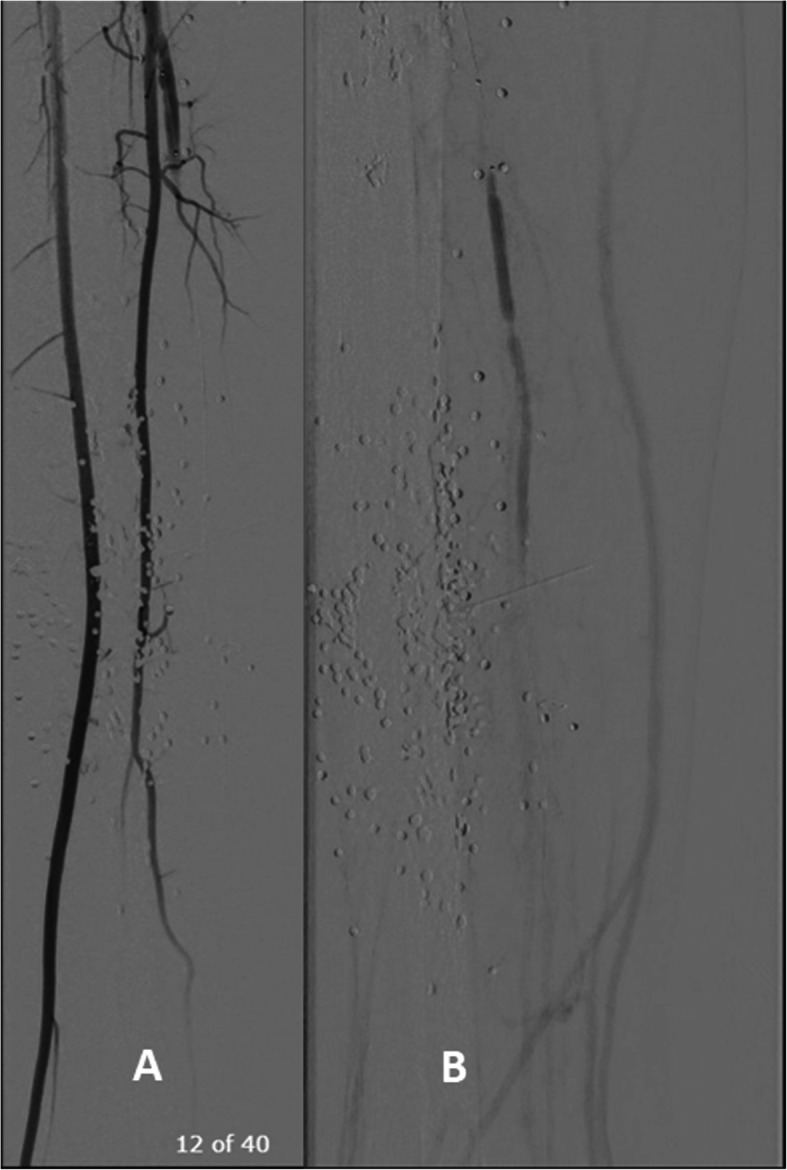
**a** Absence of AVF between PTA and PTV post stenting. **b** Sluggish flow but patent PTA

The endovascular procedure was as follows:

- A 6-French (Fr) sheath (Cook, Indiana, USA) was placed in the distal popliteal artery followed by a guidewire over a 4-Fr vertebral catheter in the PTA proximal to the AVF. The fistula was localized with severe stenosis just beyond it (Fig. 3).
- A 3 × 15 mm Euphora balloon (Medtronic, Ca, USA) was used for pre-dilatation, followed by placement of a 3 × 18 mm Bentley stent (InnoMed, Germany) in the PTA at the level of the AVF (Fig. 4).
- Post-procedure arteriogram showed total exclusion of the AVF with a patent artery distally and a sluggish but antegrade flow that reaches the plantar arch (Fig. 5). Foot and leg were briskly perfused through patent anterior tibial and peroneal arteries.

The patient was discharged on dual antiplatelet therapy (DAPT): aspirin 100 mg and clopidogrel 75 mg. On follow-up, the patient had persistent swelling of his RLE. Duplex scan showed resolution of the distal AVF, normal venous flow in the PTV, reduced flow in the proximal PTA and strong retrograde filling of the distal PTA, which was likely due to distal stent stenosis. Moreover, the AVF between the popliteal artery and vein was well visualized this time. A month later, the patient was admitted for surgical repair of his popliteal AVF.

**Fig. 6:**
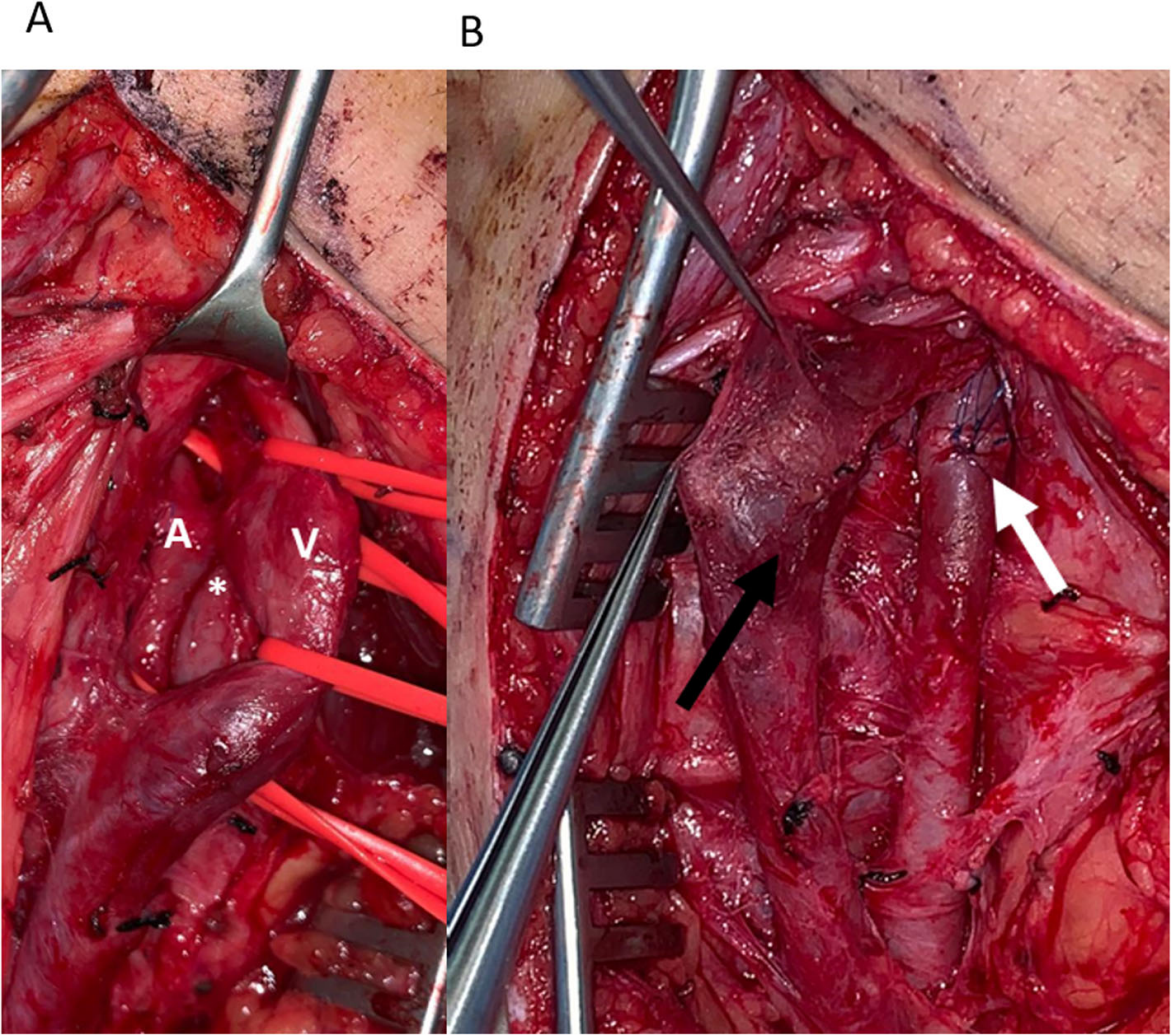
**a** Surgical exploration of the AVF (*) between popliteal artery (A) and vein (V), both of which have been controlled proximally and distally. **b** Popliteal artery and vein post debridement and AVF repair. (White arrow is arterial sutures and black arrow is venous sutures)

The surgical procedure was done through a posterior approach and a lazy S incision was performed. The short saphenous vein and sciatic nerve branches were protected. The popliteal artery and vein, which was dilated and had a thrill, were dissected and then controlled proximally and distally with vessel loops. The communication site was identified (Fig. 6 A) and both vessels were debrided and primarily repaired (Fig. 6 B). Distal pulses were palpable on completion with no thrill in the vein. The patient was discharged 2 days later on clopidogrel 75 mg and rivaroxaban 20 mg for 3 months.

At 3 months follow-up, the swelling and pain had resolved. His duplex showed no AVFs, yet it revealed an occluded right PTA at the distal stent with good reversed flow distally.

## Discussion and conclusions

TAVF is caused by penetrating trauma, blunt trauma and fractures [4–6]. In our area, which is a conflict zone, the most common etiology in our experience is shrapnel injury. TAVFs are classified into central and peripheral, the latter being more common, and among peripheral TAVFs, the lower extremity is the most frequent site [6]. Our patient developed PT and popliteal AVFs, which represent 8.6 and 1.4% respectively of traumatic fistulas [6]. TAVF manifests in three broad categories: localized early symptoms like those of our patient, such as swelling and pain [5]; local complications like arterial and venous insufficiencies [5, 7–10]; systemic complications like high-output heart failure and pulmonary hypertension [11, 12].

Table 1 summarizes the case reports and series available in the literature about TAVF. It demonstrates the wide variability of TAVF in terms of site, time to diagnosis, clinical manifestation and treatment modalities, explaining the lack of formal guidelines for diagnosing and treating TAVF.

**Table 1:** Summary of case reports and series discussed

Author	Mechanism of Injury	Location (artery)	Time to Diagnosis	Clinical Presentation	Diagnostic Method	Treatment
Ariyoshi et al. [13]	Shrapnel	SFA	2 weeks	Pseudoaneurysm	Angiography	Open surgery
Frishman et al. [14]	GSW	SFA	63 years	Arterial aneurysm, Heart failure	Angiography	Open surgery
Kim et al. [5]	Blunt injury	SFA	3 months	Iliac vein thrombosis, Venous hypertension, Pseudoaneurysm	CTA	Stent graft
Morano et al. [15]	GSW	Popliteal	1 month	Pseudoaneurysm	Angiography	Open surgery
Gorsi et al. [16]	GSW	SFA	8 years		CTA, Doppler US	Stent graft
Sahin et al. [7]	Stab wound	SFA	1 year	Claudication, Stent graft misplacement	Angiography, Doppler US	Open surgery
Roth et al. [8]	Open fracture	PTA	1 year	Non-healing ulcer, Impaired wound healing	Angiography	Open surgery
Donmez et al. [9]	GSW	PTA	10 years	Heart failure, Venous ulcers, Varicose veins	Angiography, Doppler US	Detachable balloon, NBCA
Vagefi et al. [11]	GSW	Internal iliac	40 years	Atrial fibrillation, Heart failure	CTA	Embolization, Stent graft
Veldhoen et al. [12]	GSW	Profunda femoral	6.5 years	Pulmonary hypertension	Angiography, Doppler US	Open surgery
Topuz et al. [17]	Stab wound	Profunda femoral			Doppler US	Coil embolization
Spirito et al. [18]	Blunt injury	ATA	2 months		Doppler US	Stent graft
Yilmaz et al. [19]		Internal iliac	6 years	Venous outflow obstruction		Stent graft by transvenous approach after failed open surgery
Orrapin et al. [20]	(A) Stab wound (B) Shotgun wound	(A) SFA (B) External iliac, CFA, SFA	(A) 18 years (B) 2 years	(A) Venous aneurysm causing DVT postop (B) CHF preop, Branham effect postop	(A) Angiography, Doppler US (B) CTA	(A) Open surgery (B) Open surgery
Franz et al. [21]	Stab wound	Popliteal	3 months	Stent thrombosis	CTA	Stent graft
Sahin et al. [22]	23x GSWs, 4x penetrating injuries	26x UE, 1x LE	Mean 16 ± 8 months		Doppler US	2x ligation & 1° repair of artery and vein, 5x arterial graft interposition plus 1° vein repair, 20x arterial and venous graft interposition repair

*ATA* anterior tibial artery; *CFA* common femoral artery; *CHF* congestive heart failure *CTA* Computed tomography angiography; *DVT* deep venous thrombosis; *GSW* Gunshot wound; *LE* lower extremity; *PTA* posterior tibial artery; *SFA* superficial femoral artery; *UE* upper extremity; *US* ultrasonography

Current practice considers arterial duplex scan as the initial screening method for a suspected arterial injury. It has been shown to have a sensitivity of 95% and a specificity of 98 to 99% in detecting arterial injuries [23, 24], one of which is TAVF. In our case, duplex scan detected initially one of our patient’s two AVFs. It is likely that the second one was overlooked once the fistula flow was attributed to the tibial, more distal one detected first. The popliteal fistula was readily detected on subsequent follow up, particularly following the first procedure.

There are no clear guidelines as to what the next diagnostic step is. Although angiography remains the gold standard, it is common among practitioners to do a CTA as a second step (Table 1) [16, 25]. In a systematic review and meta-analysis by Jens et al., CTA was shown to have a sensitivity and specificity of 96.2 and 99.2% respectively [26]. The lack of significant difference between the sensitivities of duplex scan and CTA decreases the utility of CTA as a second diagnostic measure after duplex scan. This is further inferred by the increased risk of artifacts in CTA due to shrapnel in the setting of a TAVF, as in the case of our patient, whose both AVFs could not be directly and accurately visualized. We have had similar experience with other blast injuries that include multiple metal shrapnel, readily detected on plain x-rays, and multiple pellets from shotguns shot at close range (thus clustered in distribution). Hence, we recommend liberal use of angiography following duplex scan rather than CTA, if the latter is not needed for other bodily injuries. This is in line as well with one of the largest series reporting on 27 operated cases of TAVF, the majority (except the ones who were unstable) had eventually angiography as a localizing modality [21].

The introduction of endovascular techniques has revolutionized vascular surgery and trauma. Unless there are restrictions like hemodynamic instability [22], patients with TAVF deserve a chance to be treated with endovascular procedures, which carry less postoperative risks and minimize magnitude of the procedure and length of stay [27]. Availability of such treatments should not compromise durability, especially in younger individuals; patient selection remains of essence. Options include AVF embolization using coils, detachable balloons or embolization glue, such as NBCA-lipiodol [9, 11, 17], and placement of stent grafts to exclude AVFs (Table 1) [5, 16, 18]. Despite the established safety of endovascular repair, patients remain at risk of detachment, embolization and stent misplacement [7, 9], as well as endoleak [16]. In a nice index case by Sahin et al. [11], surgically repairing a misplaced stent in the femoral vein across the fistula, illustrating what originally could have been a simple repair, is transformed into a complex reconstruction, especially on a young individual with low surgical risk to start with. Moreover, embolizing the feeding artery rather than the AVF if unsuccessful can prohibit or complicate further endovascular attempts [19], or force an operation.

**Fig. 7:**
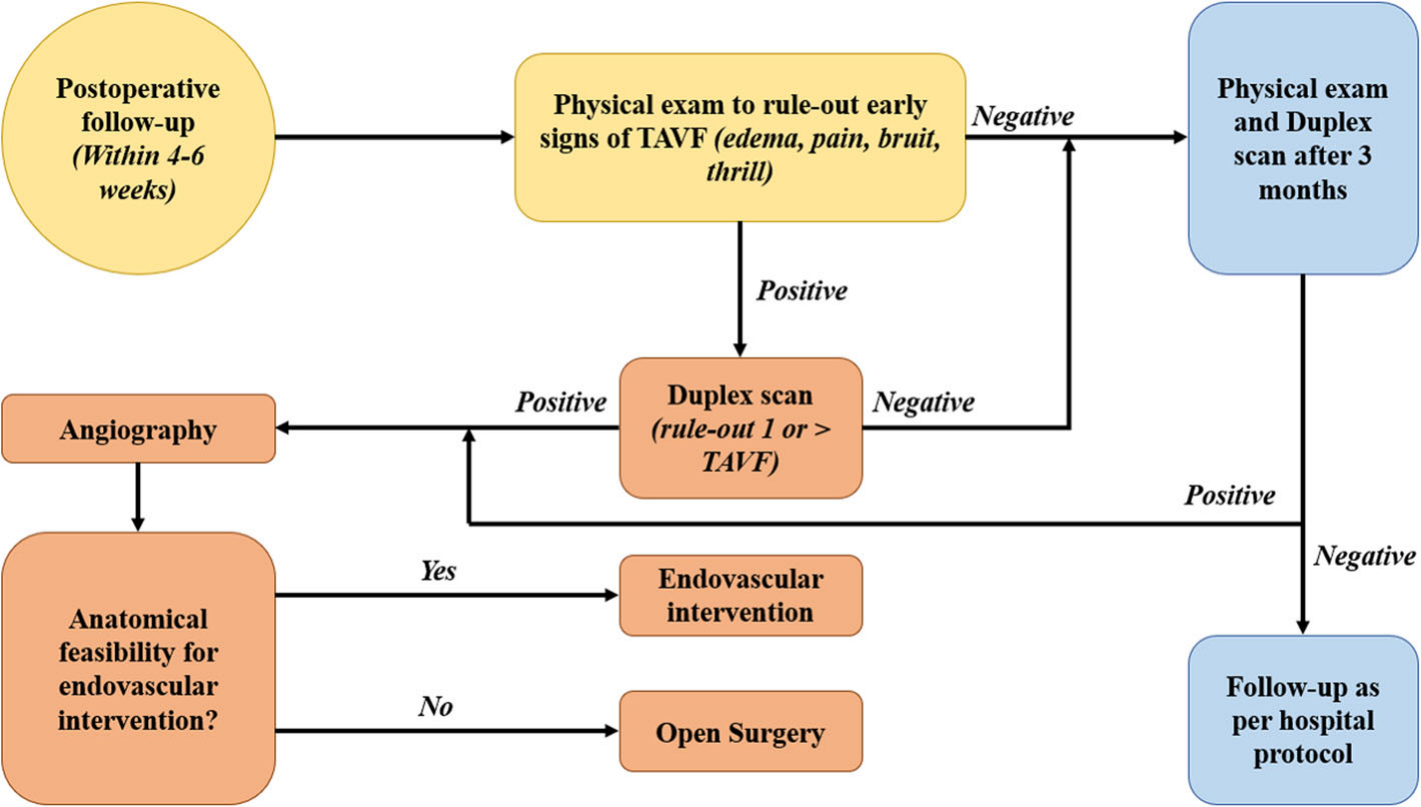
Proposed treatment algorithm for TAVF caused by pellet or shrapnel injury

In our case, we placed a stent-graft to treat the PT AVF without facing any complications. Nevertheless, in the presence of two other vessels run-off and good retrograde filling of the PTA, complete embolization of the artery would have been an option. Indeed, with the latest follow up and the occlusion at stent level, our intervention has eventually acted like an embolization procedure. It is worth noting that the early stent failure we encountered is likely due to stent sizing, which is the result of the varying PTA diameter across the fistula. Prior to angioplasty, PTA just beyond the fistula had severe stenosis, which caused our stent to be undersized, a common etiology for stent restenosis [28]. Moreover, a large number of indications in the reported surgical series were actually unsuccessful attempts at or failure of an endovascular treatment [21], speaking only of the challenging nature of those procedures and the high level of expertise to achieve technical success. Our proposed algorithm for management of pellets/shrapnels induced TAVF is summarized in Fig. 7. Concerning the sequence in dual fistulas, while some suggest repairing the larger fistulas first and leaving the smaller ones to reduce venous thrombosis [20], our situation was different since we caught the AVF rather early after the events; no venous aneurysms were present and only moderate venous dilatation. In any case, we elected to keep anticoagulation following the repair of the second, more proximal fistula for 3 months.

While cases of repairing popliteal AVFs endovascularly have been reported, we elected to surgically repair this one due to the potential increased risk of stent fracture and thrombosis across the knee joint, especially in a young active individual [28, 29]. This was the scenario in the popliteal case reported by Franz and Jump [28], especially that a stiffer stent was used originally for the repair. For us, the surgical posterior approach was the obvious choice considering the location behind the knee joint, the very focal nature and the scarring medially from the previous exploration and tissue loss.

Correction of AVFs cause rapid resolution of symptoms, including the systemic ones [11, 12, 29, 30]. There are three postoperative complications, however, that warrant special consideration; chronic TAVFs can cause venous dilatation and aneurysms, which in the setting of decreased blood velocity post repair, can precipitate the formation of venous thromboembolism (VTE) [20]. Therefore, close monitoring and DVT prophylaxis are necessary. We elected to keep our patient on anticoagulation for 3 months following the repair. The second complication is the Branham effect, which is reflex bradycardia immediately after repairing an AVF due to the sudden increase in systemic vascular resistance [20]. In case of surgical correction, Orrapin et al. recommend clamping the fistula prior to definitive excision to check for possible hemodynamic instability [20]. The third complication is aneurysmal arterial dilation proximal to AVF even post closure. Although brachial artery aneurysm is what has been reported [31, 32], the possibility of it occurring in different arteries is plausible [33]. Vigilant follow-up for new mass or swelling at site of AVF with the aid of duplex scan is the best option for early detection and treatment.

In conclusion, although TAVF is an established complication and what we are presenting is only a case report, it is an interesting illustration of this pathology that highlights the importance of vigilant follow up in pellet injuries and explores dual management options: endovascular and open repair. We recommend following up patients who sustain injuries with shrapnel or pellets with the possibility of more than one AVF in mind. Duplex scan is highly sensitive to detect those AVFs, though more than one could be present as in this case. Angiography should remain gold standard, particularly with extensive metal artefacts. Endovascular repair when feasible should be considered first, unless the patient is unstable or has anatomical constraints that increase the risk of complications (Fig. 7). Lastly, surgeons should be weary of DVT, the Branham effect and arterial aneurysmal dilation postoperatively.

## Data Availability

All data supporting the conclusions of this study are included in this published article.

## Abbreviations

AVF: Arteriovenous fistula
CFA: Common femoral artery
CTA: Computed tomographic angiography
DAPT: Dual antiplatelet therapy
DP: Dorsalis pedis
DVT: Deep venous thrombosis
Fr: French
PT: Posterior tibial
PTA: Posterior tibial artery
PTV: Posterior tibial vein
RLE: Right lower extremity
ROM: Range of motion
SFV: Superficial femoral vein
TAVF: Traumatic arteriovenous fistula
TP: Tibioperoneal
VTE: Venous thromboembolism
